# Five essential principles of post-disaster psychosocial care: looking back and forward with Stevan Hobfoll

**DOI:** 10.3402/ejpt.v4i0.21914

**Published:** 2013-08-22

**Authors:** Michel L. A. Dückers

**Affiliations:** Impact, National Knowledge and Advice Centre for Psychosocial Care Concerning Critical Incidents, Partner in Arq, Psychotrauma Expert Group, Diemen, The Netherlands

**Keywords:** Psychosocial care, disaster, community intervention, social change, interview

## Abstract

In 2007, a leading article was published by Stevan Hobfoll and a team of international experts. The authors synthesized available scientific evidence and distinguished five essential principles of psychosocial care to people confronted with disaster, tragedy, and loss. Care givers should promote: (1) a sense of safety, (2) calming, (3) self- and community efficacy, (4) social connectedness, and (5) hope. After their publication, the “essential principles” influenced the thoughts of policy makers, care providers, and scholars from all over the world. They have been embedded in several guidelines. In this interview, Professor Hobfoll is invited to revisit the principles and to look forward: “The next step is to create passageways and mutual partnerships.”

## Interview with Stevan Hobfoll, the Judd and Majorie Weinberg Presidential Professor and Chair, Rush University Medical Center, Chicago, Illinois, United States

*The last couple of years has seen many programs initiated internationally to formulate guidelines or criteria for optimal psychosocial care concerning critical incidents. The five essential principles—promote (1) a sense of safety, (2) calming, (3) self- and community efficacy, (4) social connectedness, and (4) hope (Hobfoll et al.,* *2007**)—are disseminated remarkably well among scholars. How do you view them today?*

The five principles were taken as a distillation of the literature and opinions of experts. I believe we got it right. They are the basics. In basketball the essential principles are shooting, dribbling and passing. These three are fundamental. If you want to play with the youth team, they will tell you it is all about basics, basics, basics. Special tricks come in later.

### 

#### Are the principles complete?

No, they are not and they should not be. That is to say, I really think we must *deepen* the principles, for instance by disentangling the complicated relation between religion, spirituality and meaning. I believe that spirituality matters, but there is no evidence that the religious do better. What is spirituality if you take it out from religious beliefs? Secondly, though I do think the principles are evolutionary and susceptible to gradual change, I feel we should not make *more* principles, because you then run into the resources thing – essential principles should also be applicable in poor resources contexts. Keeping the principles the way they are has another benefit. People have their own agenda, an agenda that depends on political interests. As soon as principles are expressed in terms of resources, discussions will take place that take away energy from the main issue. Delivering the five essentials to children, the elderly, situations where men and woman are different and so on, it is difficult enough. I am not saying they are complete, but it is a solid basis.

*Benedek and Fullerton wrote a reaction to the five essential principles. “The authors stop short of suggesting a means by which the elements of this framework might be advanced into practice in specific disasters or incorporated into existent public health plans. Embedded in ‘next steps’ questions are the issues of dissemination, delivery, and prioritization” (Benedek & Fullerton,* *2007**). What is your answer to them?*

They are right. Fast disasters or slow disasters like poverty are always difficult in the coalition of response action. We must keep close to the five big things, but the main challenge is in the access. The U.S. is one the wealthiest nations, one of the most resourced countries. For veterans there are 600 or 700 organizations. Yet, most of them still fail to get the care they need. To treat them right, you must know the passageways. People must be able to access resources and interventions. The accompanying piece to the original paper would have to be about passageways and obstacles.

#### Connecting people with problems to the right care. What types of obstacles do you perceive?

To stay with the previous example. Soldiers and veterans are still not taught effective PTSD approaches. There are effective treatments, and enough treatments. It is about the connection and their understanding that there is care available. We should empower the people that need care. Part of it is about the attitude of individuals and their organizations. Throughout the military soldiers were screened and treated, and told what was going to happen. When they return to the society this stops. That is a problem. Gender is also a problem. Medical establishments and organizations are led by men. Men sometimes have difficulties with emotions. Another problem is our language. All cultures have stigma in their language. It is important that we find de-stigmatizing language. Calling an illness, is part of the medical medicine. However, when 30 or 40 percent suffers, than we have a normal condition, not an exception. Let me give an example of the difficulties you run into when you want to address this problem. Some time ago we concluded that an intervention for traumatized woman is stigmatizing because it emphasizes their position as being traumatized. So we changed it. The woman treated are delighted. It [treatment] feels now like a spa, not medical. But medical professionals and managers do not want to run a spa, they want to run a medical institution. That fits with their role patterns and expectations. We really need to take a different view. That is what I try to do myself.

#### How do you do that?

When people walk into my office they say: ‘I never walked into a doctor's office that feels like home’. I want it to be a nice place. I have a lovely room with books, a couch, two comfortable chairs. It is like a living room. There is a desk where three people can sit and a coffee table. I always ask them if they want a cup of coffee, tea or water. I am serving them, which is an important entree act. You should never drink coffee alone.

*In this we can see an approach to reduce obstacles in the individual relation with patients. We have discussed several other obstacles so far. Lack of knowledge and empowerment, gender, stigma and role patterns. What about social circumstances?*

My hospital in Chicago is near West Side. It is a junction away from one of the most dangerous neighborhoods of the United States. But it is one of the top hospitals. The poor have good access, better even than the working class. It illustrates how the access and entry points of the pathways we design often are hindered by social obstacles that can be solved by what I call ‘human right attorneys’. Human right attorneys are necessary to solve situations where a person with a specific accent cannot have a job at a bank, or because of their name, color of skin or gender. If people of Moroccan descent are fifteen percent of a community and two percent of a company than it is racism. We need human right attorneys to address these issues. The challenge is to make sure people in difficult positions find equal access after a shocking event.

#### We are talking about community interventions now

Yes, rooted in the so-called Chicago School of Sociology. A century ago Jane Addams, a true believer of self-determination, gave an important impulse to the sociological research in the city, focusing on immigrants and their social-economic position. She cared for ‘her immigrants’ and only allowed researchers to collect data if they were prepared to live in the areas of the city they wished to study. The status of immigrants improved because of investments in better housing, English education, hygiene, and access to jobs. This is psychosocial care in the broad sense. In those days there were jobs you could train for easily. Such jobs are still here today. Yet, there is an obstacle. Good education for people is cheapest on long term, but expensive on the short term, and therefore not popular among politicians.

#### If we draw this line a bit further, what kind of future do you see for Europe?

Returning to the five principles, they do not speak to cultural diversity, and this has to be adapted in a culturally sensitive way. I also think that the five principles do not by themselves address situations of internal strife when the conflict is one between ethnic groups or religious groups that are living in each other's midst. We see an increase in terrorist activity, especially of radical Islamic origin imported to the West. Rightwing counter responses are very likely. I think your plate [in Europe] for the future is full. Europeans criticized Americans for a long time for having difficulties with ethnic problems. But now we have a head start. The children of second generation Muslims in America are better educated than their generation members of the traditional population. In America, let me put it simply, participation is much more accessible. Loving baseball, speaking English and saying ‘America is great’ is enough to be an American. These differences and historical trends cannot be ignored by mental health professionals, or at least we do so at our peril. They will make a difference in how we intervene, who we have to bring in to intervene, preserving integrity of subgroups, and not allowing ethnic tensions to rise to the level of pogrom or worse. Our intervention can make a difference here. So, we must add the knowledge base of other areas of community intervention to the five principles, including involving cultural subgroup representation in the entire process from planning to intervention to follow-up.

#### You are sketching a troublesome future for the old continent

While politically liberal and left myself, some liberal psychologists and psychiatrists state through a postmodern approach that we should not have nationalism. I think this is the wrong answer. People carry a nationality. It is a matter of respect to allow them to preserve their original identity. But people should develop. It is natural to adapt. You should learn new ways. The interpretation of liberalism is part of the problem. Postmodernism dislikes the idea of preserving cultures, or somehow believes that you can have culture without nation. The evidence shows that this is the wrong pattern. This leads to poverty, violence and non-acceptance. In a global situation joint ownership is necessary. The next step is to create passageways and full partnerships. Let the people we work for train us. We can learn from the positive aspect of, for instance, Islam. Take their treatment of the elderly. In India you have to kiss their feet.

#### The classical anthropological rule is to accept and not judge another's culture. How do you view this?

I must consider my values as well. I do not accept unequal treatment of women or domination of women. So, I take a stand that I may not fully accept another's culture. I will not contribute to domination and disenfranchisement of women to happening there and certainly say no to having it imported in the areas I am. As a host country I will not tolerate violence, child abuse or sexual abuse. So, I take a stand and say, ‘In your country this may be okay or tolerated. In my country [the U.S.A.] it is not.’

Postmodernism can be a rather elitist of philosophy. Nationalism is a good thing. The problem is that we have to formulate ‘inclusive’ and not ‘exclusive’ nationalism. Violence, exploitation, war are not necessary outgrowths of nationalism, and often exist more when we strip people of tribal or national heritage. We humans have tribal sentiments in our biology. You know you are doing well if the immigrants are rooting for their adopted country's national team in the World Cup. The immigrants should be just as proud when the Dutch team beats the German team as all the Dutch are.
